# Surgery

**Published:** 1903-09

**Authors:** T. Carwardine


### SURGERY.

**Primary Carcinoma of the Vermiform Appendix.**—Dr. A. W. Elting[5] has looked up the original reports of recorded cases of carcinoma of the vermiform appendix, and finds that although doubtful cases were described by Merling in 1838,[1] and by Prus in 1865, the first important article on the subject was by Rokitansky in 1867, who reported four cases of what he believed to be primary carcinoma of the appendix.[2] The first case was observed in 1847 in an individual 82 years of age. In this and in the other three cases the walls of the appendix were converted into a fibrous sac, from which trabeculæ extended into a lumen containing greyish gelatinous material; but in the absence of a microscopical report these cases may be considered somewhat dubious. He examined the literature of reported cases up to 1902, and he found that of thirty-two reported cases of primary carcinoma of the appendix, twenty could be regarded as proven and confirmed by microscopical examination. To these Elting adds three cases studied by himself in patients aged 81, 36, and 19 respectively. One showed the structure of an alveolar carcinoma arising in an obliterated appendix, and in the other two the carcinoma was colloid, in one of them the colloid contents projecting through an aperture opposite the mesenteric attachment.

Carcinoma of the appendix seems to be one of the sequelæ of chronic inflammation of the organ, exemplified by its occurrence in an obliterated appendix; and seeing that the carcinomatous nature of the affection has usually been accidentally discovered on microscopical examination, and not suspected before, it is probable that a more careful routine examination of appendices after removal for inflammation would result in a greater number of cases of carcinoma of the appendix being discovered.

The early age at which the disease may be found is a matter of interest and importance. It has been found in a child of 12. In 24 per cent. of the cases the patients were under 20, and in 53 per cent. were under 30. It will be observed that in respect of the early age of the patients the relationship to appendicitis is very close.

The colloid type of cancer seems to predominate, and in a considerable number of cases the disease is entirely confined to the appendix. He draws the following conclusions from this study: (1) Primary carcinoma of the appendix is not so rare as hitherto supposed; (2) Appendices should be examined microscopically after removal as a routine; (3) The relationship to chronic appendicitis is intimate; (4) It tends to appear early in life; (5) It does not show a marked tendency to metastases; (6) The symptoms are the same as those of chronic appendicitis; (7) The clinical diagnosis is usually impossible.

Alexis v. Moschowitz[3] has had occasion to operate on three cases of undoubted carcinoma of the appendix during the last eighteen months, and gives illustrations of the macroscopical and microscopical appearances. The first patient, aged 37, had

[1] *J. de l'Expérience*, 1838.

[2] *Wiener med. Wchnschr.*, 1866, xvi. 863. [3] *Ann. Surg.*, 1903, xxxvii. 891.

suffered from gastric attacks, and recently from frequent severe attacks of abdominal pain, culminating in an attack of suppurative peritonitis demanding operation. The tip of the appendix showed a carcinomatous mass the size of a bean, the gastric attacks being proved to arise from benign stenosis of the pylorus.

His second case was that of a woman aged 20 with a few days' history of appendicitis, and after extirpation of the appendix it was found to contain a hard nodule of carcinoma an inch from its tip. The third case was that of a woman aged 24, five months pregnant, with hypogastric pain and tenderness most marked on the right side. On operation, the appendix presented the slight anomaly of being adherent to the iliac fossa, and the other organs were normal. The appendix contained a carcinomatous mass in its obliterated distal end.

He agrees with Dr. Elting in having found about twenty authentic cases already recorded of carcinoma of the appendix, and he concludes that it must be more frequent than supposed, notwithstanding the fact that Nothnagel and Maydl found only two cases of primary cancer of the appendix in over forty thousand autopsies. Only fourteen of the previously recorded cases are available for clinical study, and the symptoms may be classified as follows: *Pain* and *tenderness* were present in three-fourths of the cases, but any suggestion of a mass was only present in a quarter of them, and rigidity in a like number; the *temperature* and *pulse* afford no deductions of any value; *adhesions* were present in half the cases, and a history of *previous attacks of inflammation* in some 60 per cent. In this connection reference is made to the observation of Letulle and Weinberg of carcinoma in two cases of "obliterating appendicitis."[1] As regards *age*, two-thirds of the patients were below 30, the majority occurring in the second and third decades, when inflammations of the appendix are most frequent—a fact confirmed by the constant presence of inflammatory signs in a carcinomatous appendix. Females were more liable to it than males in the cases already reported.

The *pathological appearances* varied, and there was no relationship between the length of the appendix and the development of cancer. The sizes of the cancerous nodules varied from one merely microscopical to one the size of a marble; but it is well for the reader to be somewhat sceptical in the diagnosis of cancer which is nothing more than microscopical! And it was only in studying this question that the writer's attention was drawn to the work of Askanazy, "wherein he calls attention to the inflammation of the lymphatic vessels which accompany the sympathetic nerves, in inflammatory diseases of the peritoneum and intestines. We find in these cases the perineural lymph spaces more or less filled with cells, forming a picture not unlike that of a carcinoma alveolus." As to the variety, most of the examples appear to be adeno-carcinomata, the majority encap-

[1] *Arch. d. Sc. méd.*, 1897.

suled or well-defined, and in some cases, as in one described by the writer, not even involving the musculature. It is variable in position, but usually located towards the distal end. It invariably appears first in the mucous membrane, infiltrating the other coats of the appendix in succession. Some inflammation or obliteration almost always accompanies the carcinoma, and the inflammation may have led to perforation or gangrene.

Moschowitz arrives at the following conclusions, which might be compared with those of Elting already given: (1) No exact figures can be given regarding the frequency of carcinoma of the appendix: it is rare, but with increasing watchfulness more cases are likely to be discovered; (2) "Primary" carcinoma of the appendix always begins in the mucosa, and probably (3) takes origin in preceding inflammation; (4) It occurs at an early age, when inflammatory processes are more common; (5) It occurs in the female more frequently than in the male in the proportion of 3 to 1; (6) The possible advent of carcinoma is an additional argument for the removal of an inflamed appendix.

**The Operative Treatment of Facial Paralysis.**—Whether arising from traumatism during operations upon the middle ear or from other causes of inflammation of the facial nerve in the Fallopian aqueduct, it is fortunately the rule for signs of recovery from paralysis to appear within six months of the onset of the trouble. But the exceptional cases in which the paralysis remains permanent have excited more surgical interest of late from the demonstration to the hilt by Messrs. Ballance and Stewart [1] of the part played by the peripheral segment in the regeneration of divided nerves, and from the greater frequency of facial paralysis arising from life-saving operations upon the mastoid region, even when it is certain that there has been no direct injury to the nerve, and the paralysis has not appeared till some hours or days after operation.

A successful case is described and freely illustrated by Harvey Cushing,[2] the proximal end of the divided spinal accessory nerve being united to the distal end of the paralysed facial nerve, and at the time of writing—six months after suture—a condition approaching the normal had been established. The operation was undertaken from the knowledge that successful crossing of flexor and extensor nerves had occurred in experiments on animals, and that not only may co-ordination be thus re-established, but nerves originally subserving entirely different functions, as the sympathetic and pneumogastric, may be successfully crossed, as demonstrated by Langley. The operation for the relief of facial paralysis seems to have been first performed by Faure.[3] The disadvantages of this form of anastomosis are obvious, in the drooping of the shoulder with

[1] *The Healing of Nerves*, 1901. [2] *Ann. Surg.*, 1903, xxxvii. 641.
[3] *Gaz. d. Hôp.*, 1898, 259. *Vide infra.*

some atrophy of the sterno-mastoid, and in the resulting co-ordination of facial movements with those of the shoulder. Said the patient after the operation: "When I wish to laugh straight, I can help it out with my shoulder." This is on a par with the case of the lady who was compelled to keep her arm fixed when walking, lest the co-ordinate expressions of her face, as she walked along, should be mistaken in their intent. But it is probable that there may be a return of power over individual groups of muscles, isolated from movements of the shoulder; thus Cushing's patient was eventually able to close the eyes and to whistle without elevating the shoulder at the same time. The paralysis resulting from division of the spinal accessory may be obviated by grafting a slip only of the nerve into the facial, as carried out by Faure; by performing lateral implantation of the distal facial segment into the spinal accessory, as in the operation of Kennedy; or by a simple lateral union of the two nerves, as in the method adopted by Ballance.

The co-ordinate shoulder movements and the wasting of shoulder muscles, as the case may be, have led Ballance to suggest an anastomosis with the hypoglossal instead of the spinal accessory.[1] The case of Faure was anticipated by Ballance by an operation performed at St. Thomas's Hospital in 1895. The condition seven years after was interesting; for although there is still apparent flaccid facial palsy, yet the raising of the shoulder against resistance brings out marked contraction of the apparently paralysed side of the face. The conclusions arrived at in this paper are as follows: (1) Peripheral facial palsy is remediable by facio-accessory anastomosis, but the extent of the recovery appears to be limited to associated movements in conjunction with the shoulder: in most cases the previous deformity disappears when the face is at rest; (2) For these reasons facio-hypoglossal anastomosis is suggested for future use; (3) The cases suitable for operation are those in which the paralysis has lasted so long that no recovery is to be expected, say six months without any sign of recovery; (4) A suppurative causal condition producing an infective neuritis renders the prognosis after operative treatment less favourable than after trauma. One operation of facio-hypoglossal anastomosis has been already done, but it is too early to determine the results as yet. Professor Schäfer has suggested the possibility of utilising the glosso-pharyngeal, but the nerve is too small to afford a prospect of good results in practice.

T. CARWARDINE.

